# A cross-sectional study of relationships between periodontal disease and general health: The Hitachi Oral Healthcare Survey

**DOI:** 10.1186/s12903-021-01990-6

**Published:** 2021-12-15

**Authors:** Shinsuke Kataoka, Mitsuo Kimura, Tsuguno Yamaguchi, Kenji Egashira, Yu Yamamoto, Yasushi Koike, Yuki Ogawa, Chika Fujiharu, Toshiko Namai, Kanako Taguchi, Momoko Takahashi, Asami Kameda, Tomoka Kasen, Asami Hano, Konomi Kubota, Masayuki Sato, Hiroaki Yamaga, Kaori Nohara, Mikiko Shirasawa, Chika Sekine, Maki Fukuda, Arisa Aoki, Yurina Takeuchi, Misaki Mugiyama, Kenta Mori, Keigo Sawada, Yoichiro Kashiwagi, Masahiro Kitamura, Takeshi Hayashi, Tohru Nakagawa, Shinya Murakami

**Affiliations:** 1 Research and Development Head Quarters, LION Corporation, Odawara, Kanagawa Japan; 2 The LION Foundation for Dental Health (Public Interest Incorporated Foundation), Sumida, Tokyo Japan; 3 Hitachi Health Care Center, Hitachi Limited, Hitachi, Ibaraki Japan; 4 Faculty of Dentistry, Osaka University, Suita, Osaka Japan

**Keywords:** Cross-sectional, Periodontal disease, Occlusal force, General health, Health check

## Abstract

**Background:**

This cross-sectional study performed to clarify the relationship between periodontal disease and non-communicable diseases (NCDs), such as obesity, diabetes mellitus, impaired glucose tolerance (IGT), chronic obstructive pulmonary disease (COPD), and atherosclerotic cardiovascular disease (ASCVD) by introducing dental examinations into the annual health examinations conducted by Japanese companies, and to highlights the importance of a medical system that connects dental and medical professionals.

**Methods:**

A total of 1.022 Hitachi Ltd. employees were enrolled in this cross-sectional study. We examined correlations and odds ratios (ORs) between the dental and overall health of employees using stratification and multiple logistic regression analyses based on the periodontal health indicators, general health indicators, and occlusal force.

**Results:**

The adjusted OR of PPD for obesity (OR, 1.42; 95% confidence interval [CI], 1.09–1.84; *p* = 0.009), IGT (OR, 1.48; 95% CI, 1.00–2.20; *p* = 0.049), and COPD (OR, 1.38; 95% CI, 1.02–1.88; *p* = 0.038) significantly differed. The adjusted OR of body mass index (OR, 1.28; 95% CI 1.15–1.42; *p* < 0.001), haemoglobin A1C (HbA1c) (OR, 4.34; 95% CI, 1.89–9.98; *p* < 0.001), fasting blood glucose (FBG) levels (OR, 1.08; 95% CI 1.04–1.11; *p* < 0.001), postbronchodilator forced expiratory volume in one second/forced vital capacity ratio (%FEV_1_) (OR, 0.95; 95% CI 0.91–1.00; *p* = 0.031) and smoking (OR, 2.32; 95% CI 1.62–3.33; *p* < 0.001) for severe periodontal disease also significantly differed. Occlusal force was significantly reduced in employees aged 50–59 years compared to those aged 40–49 years. Both PPD, HbA1c, FBG levels were significantly associated with occlusal force among employees with moderate/severe periodontitis. PPD was significantly associated with occlusal force among employees with and moderate COPD, and ASCVD. %FEV_1_ was significantly associated with occlusal force among employees with IGT.

**Conclusions:**

This cross-sectional study revealed mutual relationships among periodontal disease, NCDs, and occlusal force on Japanese corporate workers. We demonstrated that a comprehensive, regional healthcare system centred on annual integrated dental and physical health examinations in the workplace will benefit employees and positively impact corporate health insurance.

**Supplementary Information:**

The online version contains supplementary material available at 10.1186/s12903-021-01990-6.

## Background

Periodontal disease is an inflammatory state caused by intra-oral bacteria. Progressive periodontal disease is accompanied by chronic inflammation that destroys the periodontal tissue supporting teeth, which can lead to tooth loss [1]. In 2010, severe periodontitis was the sixth-most prevalent health condition, affecting 10·8% of people, or 743 million, worldwide [2]. Thus, the importance of oral care centered on disease prevention and early treatment is recognized globally.

Periodontal disease might be involved in the progression of non-communicable diseases (NCDs), such as cancer, diabetes, circulatory diseases, chronic respiratory diseases, and even Alzheimer disease [3, 4]. Particularly, many studies have suggested its bidirectional association with diabetes. A study of Pima Indians with a high frequency of type 2 diabetes found a 2.6-fold higher incidence of periodontal disease among those with diabetes than those without [5]. Furthermore, poor glycaemic control increases the risk of alveolar bone resorption [6]. Obesity, which is closely related to diabetes, is a chronic inflammatory disease characterized by constant oxidative stress. Elevated fatty acid levels increase oxidative stress in monocyte macrophages, dysregulating the production of adiponectin and other adipocytokines [7]. These inflammatory- and lipid peroxidation-related diseases also increase susceptibility to bacterial infections and might promote the progression of periodontal disease [8]. However, most epidemiological studies have been conducted in countries other than Japan; thus, the diabetes and obesity metrics indicated by these studies might not necessarily represent a comprehensive global perspective, given the differences in standards of disease severity among races [9, 10]. For example, the proportion of Asians with a body mass index (BMI) ≥ 30 kg/m^2^ (defined as obese in some European countries) is low [10]. Obesity is defined at a relatively lower threshold of BMI ≥ 25 kg/m^2^ by the Japan Society for the Study of Obesity because obesity complications occur at a lower BMI in the Japanese population than in the European and North American populations [11].

Given the relationship between periodontal disease and general health, we hypothesized that inflammation caused by periodontal disease may affect general health. Moreover, the decrease in occlusal force may affect general health through changes in food intake. Occlusal force has been shown to be directly related to masticatory function and food selection. In fact, studies involving older Japanese adults have reported that a decrease in occlusal force resulted in a decrease in masticatory force [12], and that occlusal force was significantly correlated with vitamin and dietary fibre intake [13] Furthermore, increased PPD due to worsening periodontal disease has been shown to be negatively correlated with occlusal force and involved in food acceptability [14].

Clarification regarding the association between periodontal disease and NCDs highlights the importance of a healthcare system that connects dental specialists with medical professionals who interact with patients. An oral health care system that establishes better oral health by examining, finding, and treating periodontal disease as well as by promoting maintenance and improvement of general health would be ideal. Systems that include a dental assessment as part of general health examinations in the UK are promising [15]. Japan has a unique healthcare system that mandates annual health checks for all employees. However, this system does not always include dental examinations [16].

This cross-sectional study aimed to determine whether periodontal disease correlates with NCD-centred health diagnostic indicators and to determine the effect of NCDs on occlusal force. In addition, the significance of introducing dental examinations into routine health checks under the corporate healthcare system is discussed. This could serve as a means of building a comprehensive, regional, oral healthcare system at facilities that comprehensively manage and support employee health and help prevent early periodontal disease, as well as maintain and promote good general health.

## Methods

### Study design and population

A cross-sectional study was performed from 2014 to 2017 at the Hitachi Health Care Center (HHCC, Hitachi, Ibaraki, Japan), and data were collected for one week in December of each year. Written informed consent was obtained from all employees before participation in this study. The study was called the Hitachi Oral Healthcare (HTC-OHC) Survey. The Ethics Review Board at Hitachi Ltd. approved this study (approval No. 2014-58), which was conducted in accordance with the Strengthening the Reporting of Observational studies in Epidemiology (STROBE) guidelines [17]. Dental examinations were introduced as part of the regular annual Hitachi Ltd. employee health checks (about 80 employees/day) at HHCC.

### Periodontal examination

The dental examinations included probing pocket depth (PPD), bleeding on probing (BOP), number of teeth. The periodontal disease screening in this study was conducted by 15 dental hygienists belonging to the LION Foundation for Dental Health (LDH) and 5 periodontists belonging to the Department of Periodontal Disease Treatment, Faculty of Dentistry, Osaka University. Measuring periodontal disease indicators PPD and BOP is imperative to verify the relationship between periodontal disease and general health. These indicators were measured by a dental hygienist. The PPD and the BOP were measured at the disto-, mid-, and mesio-buccal, as well as the disto-, mid-, and mesio-lingual buccal surfaces of all the teeth by using a CP-12 colour coded probe (Hu-Friedy Mfg. Co. LLC., Chicago, IL, USA). This study was conducted in addition to the health examinations for Hitachi employees at the Hitachi Health Care Center from 2014 to 2017. Approximately 80 employees/day undergo a health check-up, making it necessary to accurately measure the PPD of all teeth in a short time. Therefore, the dental hygienists who performed the measurement were trained by periodontists in performing the PPD measurement method to improve the reliability of the measurement technique and eliminate bias before conducting the study. The training was conducted for one month to reach a probe pressure of 25 g using a dental probe and a microweight scale used for measurement. In addition, the PPD was measured in five volunteers belonging to Lion Corporation, and intra- and inter-examiner calibration was performed. Calculated kappa statistics resulted in a range of values from good (0.7) to excellent (0.8).

### Data collection

Health data were obtained from annual health checks conducted at the HHCC. The 47 endpoints determined at this facility included height, weight, abdominal circumference, hearing, vision, blood pressure, blood flow, electrocardiography, and other blood tests as part of the routine physical examinations. Indices for obesity, diabetes, impaired glucose tolerance (IGT), chronic obstructive pulmonary disease (COPD), atherosclerotic cardiovascular disease (ASCVD) were calculated from test results anonymized for use as a general health index. Periodontal disease indicators (number of teeth, PPD, BOP sites, periodontal epithelial surface area [PESA], periodontal inflamed surface area [PISA]), and occlusal force were also measured during dental examinations. Detailed descriptions of measurements and calculations for each indicator are described below.

### Periodontal disease index and pathophysiological classification

Periodontitis was classified based on the consensus report of the 2017 World Workshop on the Classification of Periodontal and Peri-Implant Diseases and Conditions [18]. We did not measure clinical attachment level (CAL), but classified periodontitis stages from maximal PPD values. We compared employees without PPD ≥ 4 mm and haemoglobin A1C (HbA1c) level < 5.7 (healthy) with those who had mild (stages I and II: maximum PPD ≥ 4 mm, but < 6 mm) or moderate/severe (stages III and IV: maximum PPD ≥ 6 mm) periodontitis. We calculated the PESA and PISA to quantify pocket epithelium surface areas and periodontal inflammation loads [19] using Microsoft Excel [20].

### Assessment of occlusal force

We measured bite force of full dental arches was using Dental Prescale Type-R 50H 97-μm-thick pressure-sensitive sheets (Fujifilm, Tokyo, Japan) and visualised using an Occluzer FPD-707 scanner (Fujifilm) [21, 22]. Participants maintained maximal clenching in the intercuspal position for 3 s, with the pressure-sensitive film placed between the maxillary and mandibular dental arches [23]. The Dental Prescale sheet was set up for six dental hygienists (CF, TSN, KT, MT, AK, TK). During the measurement, the centre of the Dental Prescale sheet was aligned with the centre of the participant’s jaw to cover the entire dentition according to the method of Kumagai et al. [24]. When the dental hygienist calibrated the PPD measurement, we also conducted training on the use of the sheet and setup. Occlusal force (N) data were obtained from 764 employees, and then analysed.

### Obesity index and pathological classification

Obesity was defined as BMI ≥ 25 kg/m^2^ according to the Japan Society for the Study of Obesity [11]. Participants were classified as healthy (18.5–24.9 kg/m^2^), underweight (< 18.5 kg/m^2^), or obese (≥ 25 kg/m^2^). A stratified analysis revealed that among participants considered as healthy based on BMI, those with HbA1c level < 5.7% were considered healthy in analyses.

### Diabetes indicators and pathological classification

Diabetes was defined by HbA1c levels according to the American Diabetes Association [25]. HbA1c was measured using high-performance liquid chromatography (HLC723-G11, TOSOH Co. Ltd., Tokyo, Japan). Individuals with HbA1c level ≥ 6.5%, 5.7%–6.4%, or < 5.7% were classified as having confirmed, suspected (borderline), or no diabetes mellitus (healthy), respectively.

### Blood glucose index and pathophysiological classification

Plasma glucose (mg/dL) was measured enzymatically (GA09 II, A&T Co. Ltd., Tokyo, Japan). Levels of fasting blood glucose (FBG) < 100, 100–125, and ≥ 126 mg/dL were classified based on the guidelines of the American Diabetes Association [26] as being normal, impaired fasting glucose (IFG), or impaired glucose tolerance (IGT), respectively.

### Chronic obstructive pulmonary disease index and pathophysiological classification

Lung function was assessed as postbronchodilator forced expiratory volume in one second (FEV_1_) and forced vital capacity (FVC) by using a spirometer (SP-770COPD, Fukuda Denshi Co. Ltd., Tokyo, Japan). The FEV_1_/FVC ratio (%FEV_1_) was also calculated for classification analyses [27]. Levels of COPD taken as %FEV_1_ according to the Global Initiative for Chronic Obstructive Lung Disease (GOLD) guidelines [27] were determined as mild (≥ 80%), moderate (50%–79%), severe (30%–49%), or very severe (< 30%). Participants with HbA1c level < 5.7% classified as mild by %FEV_1_ served as the healthy group during stratification analyses.

#### Atherosclerotic index and pathophysiological classification

The cardio-ankle vascular index (CAVI) was measured to determine arterial stiffness that reflects the elastic properties of arterial walls [28]. CAVI was measured using the VaSera VS-3000 (Fukuda Denshi Co. Ltd., Tokyo, Japan). Values of < 8.0, 8.0–8.9, and ≥ 9.0 were defined as healthy, borderline, and suspected atherosclerosis, respectively. Participants with CAVI < 8.0 and HbA1c level < 5.7% were defined as healthy in the stratified analysis.

#### Classification of smoking status

Smoking status was categorized as having never smoked (never), previously smoked (former), and currently smoked (current) based on questionnaire responses.

#### Statistical analyses

Pathophysiology was stratified based on the indicators of periodontal disease, obesity, diabetes, COPD, ASCVD. Differences between dental hygiene and health diagnostic indices were analysed using one-way ANOVA with post hoc Dunnet’s tests, chi-squared tests, Wilcoxon rank-sum tests, or Kruskal–Wallis tests with post hoc Bonferroni correction for multiple comparison. Differences in indices between disease and healthy groups were analysed using Mann–Whitney *U* tests. Associations between smoking status and disease states were also analysed using Pearson’s chi-squared tests (categorical variables).

The risk of developing obesity, diabetes, IGT, COPD, and ASCVD with moderate/severe periodontitis, was estimated through multiple logistic regression analyses that included dental indices (tooth count and PPD, PESA, and PISA) and health diagnostic indices (BMI, HbA1c, FBG, %FEV_1_, and CAVI). Model 1 (M1) is the reference model, which is based on healthy participants with HbA1c level < 5.7. Model 2 is M1 adjusted for age. Model 3 is M1 adjusted for age and smoking status. The odds ratios (ORs) and 95% confidence intervals (CI) between disease and control groups were then analysed. Associations between periodontal disease indices, general health indices, and occlusal force were clarified using multiple linear regression analyses. All data were statistically analysed using SPSS Statistics Version 27.0 (IBM Corp., Armonk, NY, USA). Values with *p* < 0.05 were considered statistically significant.

## Results

**Fig. 1:**
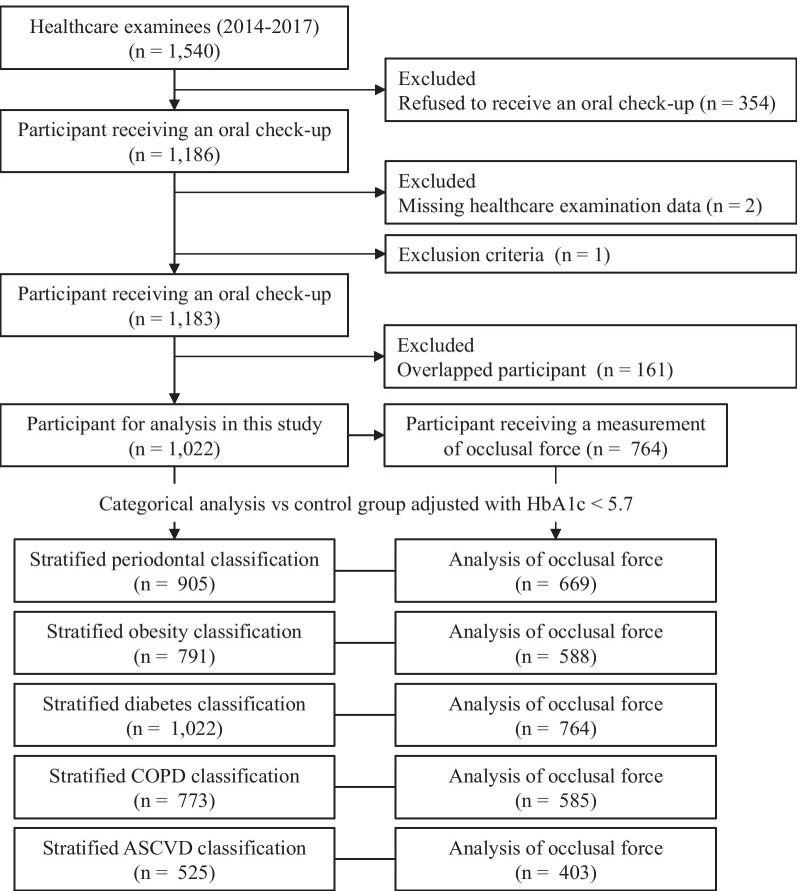
Flowchart of study participants in the HTC-OHC survey

All participants were aged ≥ 20 years who confirmed their intention to participate in the study through writing. Those who are taking medication or undergoing treatment for systemic disease and those with full dentures were excluded upon participant recruitment. Upon applying the exclusion criteria, we targeted a total of 1,183 people: 248 in 2014, 294 in 2015, 322 in 2016, and 319 in 2017. However, there was an overlap in the participants because 161 participants had already been examined; as such, the duplicate data were deleted. The final number of participants was 1,022 (Fig. 1). Table 1 shows baseline characteristics of participants in this study. Median age was 50 (28–77) years, consisting of 914 male (89.4%) and 108 female (10.6%), among whom 30% smoked and 40% did not. The median of number of teeth, levels of BMI, and HbA1c were 28, 23.7 kg/m^2^, and 5.6%, respectively.

**Table 1:** Characteristics of the study participants

*n* [Male / Female]	1022 [914 / 108]
Age [years]	50 (14)
Number of teeth	28 (4)
BOP [sites / mouth]	11 (22)
Biting force [N]	368.2 (329.7)
Hight [cm]	170.0 (9.6)
Body weight [kg]	68.7 (15.1)
BMI [kg/m^2^]	23.7 (4)
HbA1c [%]	5.6 (0.5)
FBG [mg/dL]	104 (15)
% FEV_1_	80 (8)
CAVI	7.80 (1.29)
Smoking [*n*, [%]]	
Never	406 [40]
Former	309 [30]
Current	307 [30]

Data are presented as the median (interquartile range) or number or percentage

### Classification based on periodontal disease index

Table 2 shows that 62 (6.9%) of the employees had good oral health, whereas 487 (54.2%), and 349 (38.9%) had mild and moderate/severe periodontitis, respectively. The PPD, BOP sites, PESA, and PISA were significantly increased in those with mild or moderate/severe periodontitis compared with healthy employees. In addition, mild or moderate/severe periodontitis was associated with a significant increase in BMI, FBG and HbA1c levels. The groups with moderate/severe periodontitis had significantly fewer teeth, lower high-density lipoprotein (HDL), and % FEV_1_ levels than the healthy group.

**Table 2:** Intergroup comparisons of participants stratified by periodontal status (*n* = 898)

Variables	Healthy *n *= 62 (6.9%)	Mild periodontitis (Stage I and II) *n *= 487 (54.2%)		Moderate/Severe periodontitis (Stage III and IV) *n* = 349 (38.9%)		*P* value
Periodontal parameters						
Number of teeth	27.3 (3.0)	27.1 (3.6)		25.1 (5.6)***		< 0.001^†^
PPD (mm)	2.5 (0.2)	2.7 (0.3)***		3.2 (0.7)***		< 0.001^†^
BOP sites / mouth	0.0 (0.0)	17.1 (19.4)***		28.9 (26.9)***		< 0.001^†^
PESA (mm^2^)	1277.4 (188.2)	1363.9 (221.7)***		1568.2 (444.0)***		< 0.001^†^
PISA (mm^2^)	0.0 (0.0)	169.3 (196.1)***		393.4 (424.5)***		< 0.001^†^
Health examination						
Age	49.8 (9.2)	49.9 (9.7)		52.9 (9.9)*		< 0.001^¶^
Weight (kg)	62.8 (10.1)	69.6 (12.1)***		70.6 (12.4)***		< 0.001^¶^
BMI (kg/m^2^)	22.2 (2.5)	24.1 (3.5)***		24.5 (3.7)***		< 0.001^†^
Triglyceride (mg/dL)	103.8 (80.5)	125.8 (90.0)		132.8 (98.4)		0.070^†^
LDL (mg/dL)	123.7 (26.2)	122.8 (29.5)		119.5 (30.9)		0.239^¶^
HDL (mg/dL)	63.3 (15.4)	58.2 (15.1)*		58.6 (15.4)*		0.045^¶^
FBG (mg/dL)	98.7 (8.0)	107.7 (18.6)***		110.9 (19.5)***		< 0.001^†^
HbA1c (%)	5.44 (0.21)	5.72 (0.73)***		5.80 (0.74)***		< 0.001^†^
% FEV_1_	81.1 (6.9)	80.2 (5.8)		78.3 (7.0)*		0.023^†^
CAVI	8.0 (1.0)	7.8 (0.9)		8.0 (0.9)		< 0.044^†^
Smoking (n, (%))						< 0.001^§^
Never	38 (61.3)	207 (42.5)		104 (29.8)		
Former	13 (21.0)	143 (29.4)		118 (33.8)		
Current	11 (17.7)	137 (28.1)		127 (36.4)		
Occlusal function (Total *n* = 669)						
* n*	33	352		284		
Biting force (N)	453.1 (279.7)	411.1 (244.7)		372.9 (237.4)		0.058^¶^

Data are shown as means ± SD. *P *value was calculated from one way analysis of variance (ANOVA) ^¶^ or Kruskal–Wallis tests for continuous variables or Pearson’s chi-squared tests^§^ for categorical variables

*p < 0.05, ***p < 0.001 vs. healthy group

### Health status stratified by obesity index

Table 3 shows that 421 (53.2%), 22 (2.8%) and 384 (44.0%) employees were healthy, underweight and overweight according to obesity pathophysiology. The underweight group did not differ significantly from the healthy group in terms of periodontal disease indicators. The obese group had significantly deeper PPD, higher BOP, PESA, and PISA, and fewer teeth than the healthy group. The obese group had also significantly higher %FEV_1_ and triglyceride, LDL, FBG, and HbA1c levels, with lower HDL values.

**Table 3:** Intergroup comparisons of participants stratified by Body Mass Index (*n* = 791)

Variables	Healthy *n* = 421 (53.2%)	Underweight *n* = 22 (2.8%)	Obesity *n* = 348 (44.0%)	*P* value
Periodontal parameters				
Number of teeth	27.0 (4.2)	26.4 (3.7)	25.4 (5.8) ***	< 0.001^†^
PPD (mm)	2.7 (0.5)	2.7 (0.5)	2.9 (0.7) ***	< 0.001^†^
BOP sites / mouth	17.1 (20.8)	15.5 (17.3)	22.1 (25.3) ***	0.012^†^
PESA (mm^2^)	1392.7 (308.5)	1325.4 (267.2)	1429.8 (413.6) *	0.166^†^
PISA (mm^2^)	192.0 (284.1)	162.3 (212.6)	268.3 (350.5) ***	0.004^†^
Health examination				
Age	48.7 (9.7)	49.1 (8.0)	51.0 (9.2) **	0.003^¶^
Weight (kg)	64.4 (8.0)	47.6 (5.4) ***	80.7 (12.0)***	< 0.001^†^
BMI (kg/m^2^)	22.2 (1.7)	17.5 (0.9) ***	28.0 (3.1)***	< 0.001^†^
Triglyceride (mg/dL)	109.8 (78.2)	69.3 (29.2) **	150.5 (98.9)***	< 0.001^†^
LDL (mg/dL)	118.7 (28.0)	107.1 (37.3)	127.1 (30.1)***	< 0.001^†^
HDL (mg/dL)	63.0 (15.8)	81.8 (21.8) ***	51.7 (11.1)***	< 0.001^†^
FBG (mg/dL)	100.9 (8.3)	97.6 (10.1)	115.5 (24.6) ***	< 0.001^†^
HbA1c (%)	5.37 (0.20)	5.59 (0.29)***	5.99 (0.92)***	< 0.001^†^
% FEV_1_	79.0 (6.9)	82.9 (8.3)*	80.6 (5.4) **	0.001^†^
CAVI	7.8 (0.9)	8.1 (0.8)	7.8 (0.9)	0.426^¶^
Smoking (n, (%))				< 0.001^§^
Never	200 (47.5)	10 (45.5)	117 (33.6)	
Former	120 (28.5)	2 (9.1)	104 (29.9)	
Current	101 (24.0)	10 (45.5)	127 (36.5)	
Occlusal function (Total *n* = 588)				
n	316	12	260	
Bite force (N)	410.5 (243.9)	426.1 (307.1)	395.5 (240.5)	0.727^¶^

Data are shown as means ± SD. P value was calculated from one way analysis of variance (ANOVA) ^¶^ or Kruskal–Wallis tests for continuous variables or Pearson’s chi-squared tests^§^ for categorical variables.

*p < 0.05, **p < 0.01, ***p < 0.001 vs. healthy group

### Health status stratified by diabetes index

Table 4 shows 582 (56.9%), 353 (34.5%), and 87 (8.5%) employees were healthy, borderline diabetic, and diabetic according to the diabetes pathophysiology classification index. The diabetes group had fewer teeth and deeper PPD than the healthy group. They were also heavier and had a higher BMI and higher triglyceride, FBG, and CAVI levels, but lower HDL levels and occlusal force.

**Table 4:** Intergroup comparisons of participants stratified by HbA1c (*n* = 1022)

Variables	Healthy *n* = 582 (56.9%)	Borderline *n* = 353 (34.5%)	Diabetes *n* = 87 (8.5%)	*P *value
Periodontal parameters				
Number of teeth	26.7 (4.6)	25.8 (5.2)***	23.7 (6.5)***	< 0.001^†^
PPD (mm)	2.8 (0.6)	2.8 (0.6)	3.0 (0.6)***	0.005^¶^
BOP sites / mouth	18.0 (21.0)	19.9 (24.8)	19.9 (24.2)	0.415^†^
PESA (mm^2^)	1402.7 (327.5)	1405.0 (396.8)	1347.2 (410.2)	0.465^†^
PISA (mm^2^)	206.0 (283.2)	239.9 (348.4)	251.5 (351.1)	0.202^†^
Health examination				
Age	49.0 (9.7)	53.2 (9.4)***	56.0 (9.2)***	< 0.001^¶^
Weight (kg)	67.8 (11.0)	70.5 (13.6)***	76.8 (15.3)***	< 0.001^†^
BMI (kg/m^2^)	23.4 (3.1)	24.6 (3.8)***	26.7 (4.5)***	< 0.001^†^
Triglyceride (mg/dL)	121.6 (89.4)	126.6 (80.0)	154.2 (113.5)***	0.037^†^
LDL (mg/dL)	121.1 (29.2)	123.1 (29.9)	119.2 (29.2)	0.429^¶^
HDL (mg/dL)	60.7 (16.0)	58.0 (14.7)*	52.3 (13.5)***	< 0.001^†^
FBG (mg/dL)	101.4 (8.6)	109.7 (12.9)***	154.3 (28.8)***	< 0.001^†^
HbA1c (%)	5.39 (0.19)	5.89 (0.19)***	7.70 (1.15)***	< 0.001^†^
% FEV_1_	79.5 (6.7)	79.6 (6.4)	79.5 (5.9)	0.962^¶^
CAVI	7.8 (0.9)	8.0 (0.9)***	8.4 (1.0)***	< 0.001^¶^
Smoking (*n*, (%))				0.004^§^
Never	260 (44.6)	122 (34.6)	24 (27.6)	
Former	165 (28.4)	112 (36.3)	32 (36.8)	
Current	157 (27.0)	119 (33.7)	31 (35.6)	
Occlusal function (Total *n* = 764)				
*n*	431	264	69	
Bite force (N)	416.5 (243.7)	384.1 (249.8)	334.3 (287.5)*	0.002^¶^

Data are shown as means ± SD. *P* value was calculated from one way analysis of variance (ANOVA) ^¶^ or Kruskal–Wallis tests for continuous variables or Pearson’s chi-squared tests^§^ for categorical variables

*p < 0.05, ***p < 0.001 vs. healthy group

### Health status determined based on chronic obstructive pulmonary disease index

Table 5 shows that 309 (40.0%) and 464 (60.0%) employees, respectively, had mild and moderate COPD according to the pathophysiology classification index. Fewer teeth, higher PPD, FBG, HbA1c, and CAVI, and significantly decreased occlusal force were associated with moderate COPD than with mild COPD.

**Table 5:** Intergroup comparisons of participants stratified by %FEV_1_ (*n* = 773)

Variables	Mild *n* = 309 (40.0%)	Moderate *n* = 464 (60.0%)	
Periodontal parameters			
Number of teeth	27.3 (3.8)	25.3 (5.8)	< 0.001^†^
PPD (mm)	2.7 (0.5)	2.9 (0.6)	< 0.001^†^
BOP sites / mouth	18.5 (21.0)	18.4 (22.1)	0.931^†^
PESA (mm^2^)	1412.7 (293.1)	1393.2 (412.5)	0.618^†^
PISA (mm^2^)	203.7 (260.9)	230.5 (345.4)	0.569^†^
Health examination			
Age	46.7 (8.9)	54.1 (9.4)	< 0.001^¶^
Weight (kg)	68.1 (12.0)	68.8 (11.0)	0.408^†^
BMI (kg/m^2^)	23.6 (3.5)	23.8 (3.0)	0.280^¶^
Triglyceride (mg/dL)	123.3 (89.0)	128.5 (100.4)	
LDL (mg/dL)	120.5 (28.5)	120.9 (30.4)	0.893^¶^
HDL (mg/dL)	60.7 (16.3)	59.6 (15.4)	0.333^¶^
FBG (mg/dL)	100.9 (8.8)	109.6 (19.7)	< 0.001^†^
HbA1c (%)	5.38 (0.18)	5.78 (0.73)	< 0.001^†^
% FEV_1_	84.1 (3.4)	74.0 (4.8)	< 0.001^†^
CAVI	7.6 (0.9)	8.1 (0.9)	< 0.001^¶^
Smoking (n, (%))			< 0.001^§^
Never	160 (46.5)	180 (32.8)	
Former	93 (27.0)	202 (36.9)	
Current	91 (26.5)	166 (30.3)	
Occlusal function (Total *n* = 585)			
n	218	367	
Bite force (N)	432.3 (241.2)	373.8 (238.1)	0.001^¶^

Data are shown as means ± SD. ^†^Mann Whitney U tests. Smoking data are shown as *n* (%). ^§^Pearson’s chi-squared tests

### Health  status stratified by atherosclerotic cardiovascular disease index

Table 6 shows that 245 (46.7%), 187 (35.6%) and 93 (17.7%) employees were respectively healthy, borderline, and had suspected ASCVD according to the atherosclerotic pathophysiology classification index. Those with ASCVD had fewer teeth, increased FBG, and HbA1c levels, and decreased %FEV_1_ and occlusal force. However, BMI did not significantly differ among groups.

**Table 6:** Intergroup comparisons of participants stratified by CAVI (*n* = 525)

Variables	Healthy *n* = 245 (46.7%)	Borderline *n* = 187 (35.6%)	ASCVD *n* = 93 (17.7%)	*P *value
Periodontal parameters				
Number of teeth	27.4 (6.9)	25.6 (4.8)***	23.2 (6.9)***	< 0.001^†^
PPD (mm)	2.7 (0.5)	2.8 (0.6)	2.9 (0.7)	0.016^†^
BOP sites / mouth	18.4 (22.0)	17.7 (21.0)	13.5 (16.0)	0.059^†^
PESA (mm^2^)	1399.0 (274.6)	1385.6 (346.5)	1247.8 (359.6)***	0.001^†^
PISA (mm^2^)	201.5 (262.2)	210.9 (297.7)	157.4 (195.9)	0.141^†^
Health examination				
Age	45.9 (7.7)	56.6 (8.5)***	64.2 (6.4)***	< 0.001^†^
Weight (kg)	69.1 (11.9)	66.6 (10.5)*	65.5 (9.8)*	0.009^†^
BMI (kg/m^2^)	23.6 (3.4)	23.9 (3.1)	23.3 (2.7)	0.335^†^
Triglyceride (mg/dL)	116.7 (73.6)	130.5 (108.7)	127.6 (87.1)	0.251^†^
LDL (mg/dL)	123.3 (27.9)	119.7 (28.7)	118.0 (27.0)	0.200^¶^
HDL (mg/dL)	60.4 (14.7)	58.5 (15.1)	60.7 (16.6)	0.375^¶^
FBG (mg/dL)	100.5 (8.4)	110.7 (21.2)***	118.1 (20.4)***	< 0.001^†^
HbA1c (%)	5.38 (0.20)	5.84 (0.76)***	5.95 (0.68)***	< 0.001^†^
% FEV_1_	80.2 (6.3)	79.0 (6.2)*	76.7 (7.6)***	< 0.001^†^
CAVI	7.2 (0.5)	8.4 (0.3)***	9.5 (0.5)***	< 0.001^†^
Smoking (*n*, (%))				0.001^§^
Never	110 (44.9)	70 (37.4)	36 (38.7)	
Former	65 (26.5)	69 (36.9)	45 (48.4)	
Current	70 (28.6)	48 (25.7)	12 (12.9)	
Occlusal function (Total *n* = 403)				
*n*	186	140	77	
Bite force (N)	456.3 (256.6)	357.8 (199.2)***	316.0 (200.0)***	< 0.001^†^

Data are shown as means ± SD. *P* value was calculated from one way analysis of variance (ANOVA) ^¶^ or Kruskal–Wallis tests for continuous variables or Pearson’s chi-squared tests^§^ for categorical variables

*p < 0.05, ***p < 0.001 vs. healthy group

### Factor analysis of oral and systemic health status interdependence

**Fig. 2:**
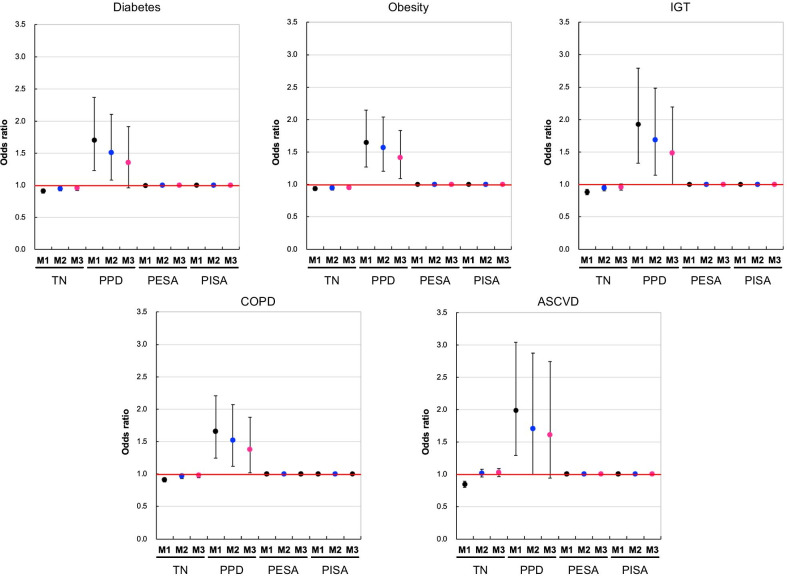
Effects of periodontal disease indicators on risk of diabetes, obesity, IGT, COPD, and ASCVD. Model 1 (M1) is the reference model based on healthy employees with HbA1c < 5.7. Model 2 (M2) is M1 adjusted for age, and Model 3 (M3) is M1 adjusted for age and smoking status. Multiple logistic regression analysis was used to obtain the odds ratio. The number of patients with disease is as follows: Diabetes; n = 87, Obesity; n = 348, IGT; n = 117, COPD (moderate); n = 464, ASCVD; n = 93. Markers indicate estimated odds ratio (OR). Vertical ranges indicate 95% confidence interval (CI).TN; Number of teeth

We examined bidirectional effects on oral and systemic health in Model 3 (reference Model 1 [M1], adjusted for age and smoking status) using multiple logistic regression analyses. Analysis of the effects of having more teeth on systemic disease revealed a significantly decreased risk of obesity (OR, 0.95; 95% CI, 0.92–0.99; *p* = 0.006), and diabetes mellitus (OR, 0.96; 95% CI, 0.92–1.00; *p* = 0.040). However, risk of the following: IGT (OR, 0.96; 95% CI, 0.91–1.01; *p* = 0.077), moderate COPD (OR, 0.98; 95% CI, 0.94–1.02; *p* = 0.249), and ASCVD (OR, 1.02; 95% CI, 0.96–1.09; *p* = 0.456) was not decreased. A deeper PPD also significantly correlated with increased risk of obesity (OR, 1.42; 95% CI, 1.09–1.84; *p* = 0.009), IGT (OR, 1.48; 95% CI, 1.00–2.20; *p* = 0.049), and moderate COPD (OR, 1.38; 95% CI, 1.02–1.88; *p* = 0.038). However, the risks of diabetes (OR, 1.36; 95% CI, 0.96–1.91, *p* = 0.083) and ASCVD (OR, 1.61; 95% CI, 0.91–2.87; *p* = 0.105) were not increased (Fig. 2).

**Fig. 3:**
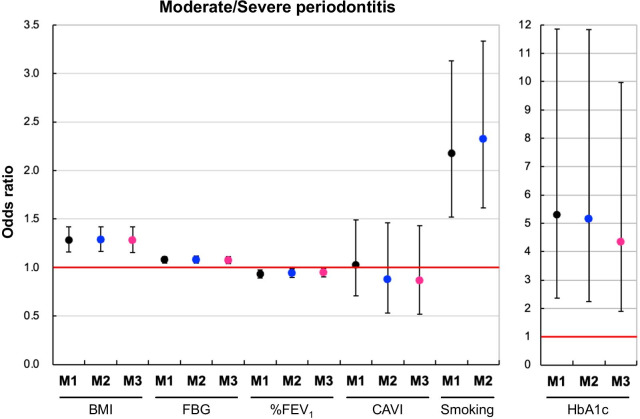
Effects of BMI, FBG, %FEV1, CAVI, smoking, and HbA1c on severe periodontal disease. Model 1 (M1) is the reference model based on healthy employees with HbA1c < 5.7. Model 2 (M2) is M1 adjusted for age. Model 3 (M3) is M1 adjusted for age and smoking status. Multiple logistic regression analysis was used to obtain the odds ratio. Severe periodontal disease; n = 349. Markers indicate the estimated odds ratio (OR). Vertical ranges indicate the 95% confidence interval (CI)

The effect of systemic disease on periodontal disease status revealed that a high BMI (OR, 1.28; 95% CI, 1.15–1.42; *p* < 0.001), HbA1c (OR, 4.34, 95% CI, 1.89–9.98, *p* < 0.001), FBG (OR, 1.08; 95% CI, 1.04–1.11; *p* < 0.001), and smoking (OR, 2.32; 95% CI, 1.62–3.33; *p* < 0.001; Model 2) significantly increased with worse periodontal disease. A low %FEV_1_ (OR, 0.95; 95% CI, 0.91–1.00; *p* = 0.031) significantly increased with worse periodontal disease. However, CAVI (OR, 0.90, 95% CI, 0.52–1.43; *p* = 0.569) did not affect risk of developing periodontal disease (Fig. 3).

### Relationship between occlusal force and general health

**Fig. 4:**
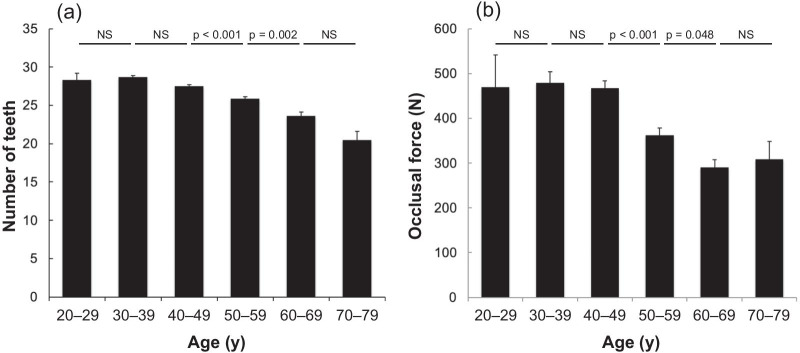
Changes in number of teeth and occlusal force with advancing age. **a** Number of teeth stratified by age (n = 1,022 employees). Mean numbers of employees in each age group were as follows: 20 s (n = 3), 30 s (n = 102), 40 s (n = 382), 50 s (n = 315), 60 s (n = 174), 70 s (n = 47). **b** Occlusal force stratified by age (n = 764 employees). Mean numbers of employees in each age group were as follows: 20 s (n = 3), 30 s (n = 83), 40 s (n = 270), 50 s (n = 241), 60 s (n = 132), 70 s (n = 35). Data are shown as means ± SE. Kruskal–Wallis test with post hoc Bonferroni test was used for multiple comparisons across the six groups. NS, not significant

Figure 4 shows age-related changes in the number of teeth and occlusal force. The number of teeth significantly decreased between 40 and 70 years of age. Occlusal force significantly decreased among employees in their 50 s (22.5% vs. age 40 s, *p* < 0.001) and 60 s (19.9% vs. age 50 s, *p* = 0.048).

We conducted multiple linear regression analysis and calculated standardized partial regression coefficients (SPRCs) (Additional file 1: Table 7) to clarify associations among the periodontal disease index, general health index, and occlusal force, as well as associations between each of these indices and occlusal force for severe pathology stratified by each index. PPD was significantly associated with occlusal force of employees with moderate/severe periodontitis (*p* = 0.001), moderate COPD (*p* = 0.008), and ASCVD (*p* < 0.001) in Model 3. Moreover, HbA1c, FBS levels were significantly correlated with occlusal force of employees with moderate/severe periodontitis (*p* = 0.022, *p* = 0.045), whereas %FEV_1_ was significantly correlated with occlusal force of employees with IGT (*p* = 0.044) in Model 3.

## Discussion

We added a dental examination to annual employee health checks at HHCC in this HTC-OHC cross-sectional survey. We assessed bidirectional relationships among the periodontal disease index and general health indices. Clarifying the association between periodontal and systemic diseases with a focus on NCDs would enable the construction of a comprehensive regional healthcare system capable of future intervention. Therefore, we examined whether an association between periodontal disease and systemic disease, which is becoming increasingly clear in many epidemiological studies, could be identified by combining the health diagnostic indices that are normally measured in annual Japanese corporate medical checks with periodontal disease indices.

Diabetes is a metabolic disorder characterized by chronic hyperglycaemia [25]. Persistent long-term hyperglycaemia causes abnormalities in various organs and complications, such as retinopathy, nephropathy, neuropathy, and cardiovascular diseases [29], and an increased risk of periodontal disease [30].

This study analysed the effects of the periodontal disease index, PPD, BOP, PESA, PISA and number of teeth on diabetes. The PPD analysis suggested that periodontal disease increases the risk of developing diabetes by 1.4-fold. Furthermore, the HbA1c findings suggested that diabetes increases the risk of developing periodontal disease by 4.3-fold, independent of age, sex, smoking. Kocher et al. summarized typical epidemiological studies of periodontal disease and diabetes, and showed an increased OR for periodontal disease risk from 1.17- to 3.84-fold due to diabetes, although a study involving Pima Indians found a 11.4-fold increase in the OR [31]. The ORs calculated herein were higher than those of previous studies. We believe that this is because we narrowed down the healthy subjects in the stratified analysis of periodontal disease. At the time of the stratification analysis, the healthy group had no PPD of 4 mm, and had HbA1c level < 5.7. Therefore, the number of periodontally healthy employees fell to 62, which might have improved the accuracy of the OR. We believe that this study also confirmed the existence of a bidirectional relationship between periodontal disease and diabetes; however, validation in a larger sample is required.

Obesity is a chronic inflammatory disease characterized by excessive fat accumulation in adipose tissues [32]. Increased oxidative stress might not only lead to local and systemic vascular endothelial failure but also to periodontal disease [33]. We found a significantly higher BMI among employees with mild and moderate/severe periodontitis than among healthy employees with HbA1c level < 5.7%. Multiple logistic regression analyses also suggested that BMI was 1.3-fold more likely to increase the risk of developing periodontitis. Conversely, having more teeth was associated with decreased risk of obesity, and that PPD was 1.4-fold more likely to increase the risk of developing obesity. The stratified analysis suggested that blood HDL level was significantly reduced in the obesity subjects (*p* < 0.001) and significantly increased in the underweight subjects (*p* < 0.001). HDL has been reported to have anti-inflammatory effects [34]. Decreased HDL levels associated with increased sensitivity toward inflammatory stimuli as reflected by enhanced inflammatory and coagulation responses on endotoxin challenge [35] *Porphyromonas gingivalis (P. gingivalis)*, a gram-negative oral anaerobe involved in the pathogenesis of periodontitis, induces oxidation of HDL [36], and lipopolysaccharide from *P. gingivalis* suppresses the secretion of adiponectin from adipocytes [37]. These studies may more accurately explain the relationship between periodontal disease and obesity.

The association between periodontal disease and COPD has given the overlap with chronic inflammatory diseases that involve the breakdown of connective tissue and the existence of common risk factors such as age and smoking [38]. We found a significantly lower %FEV_1_ among employees with moderate/severe periodontitis than among healthy employees. Multiple logistic regression analyses also suggested that COPD increase the risk of developing periodontitis. Conversely, having more teeth was associated with decreased risk of moderate COPD, and that PPD was 1.4-fold more likely to increase the risk of developing COPD. However, no differences in BOP or PISA indicators related to inflammation were observed. A meta-analysis of association studies between periodontal disease and COPD reported an increase in PPD and attachment loss in patients with COPD. However, no differences in bleeding or inflammation were reported, and no definitive results were provided [39]. The absence of a relationship between COPD and dental inflammation indices (BOP and PISA) or BMI in this study may have been influenced by the absence of subjects classified as “type III severe” or “type IV very severe” by the Gold Guideline COPD classification criteria. Therefore, further validation is required. Barros et al*.* compared COPD in toothless subjects versus those with healthy periodontal tissue (teeth and gums) and reported an elevated risk of hospitalization and death among those with toothless jaws [40]. This suggests a connection between tooth count and COPD. Early and appropriate implementation of periodontal care may reduce the risk of COPD.

The association between periodontal disease and ASCVD has been a topic of interest, given their overlap with chronic inflammatory diseases that involve the breakdown of connective tissue and common risk factors such as age and smoking [41] We found a significantly increased CAVI in employees with moderate/severe periodontitis. Adjusting for age and smoking behaviour in the multiple logistic regression analysis, the CAVI could potentially increase the risk of moderate/severe periodontitis. Conversely, employees classified as having ASCVD had significantly fewer teeth and decreased PESA compared with the healthy group, whereas PPD did not significantly differ among groups. Associations between periodontal and cardiovascular diseases have been characterized, but their causes are yet to be established [42].

The decrease in occlusal force may affect general health through changes in food intake. Occlusal force is determined by masticatory muscle strength, tooth placement, and the biomechanical characteristics of the stomatognathic system, including periodontal status [43]. It is used to evaluate the function and effectiveness of the mastication system [44]. Damaged periodontal tissues, such as deeper periodontal pockets, perturb the teeth, reducing occlusal force and masticatory ability [45, 46]. Occlusal function becomes insufficient when the mouth contains < 20 teeth, reducing maximal occlusal strength and masticatory ability [47]. We measured occlusal strength in some of the employees. Occlusal force was decreased by 22.5% and 19.9% in individuals aged in their 50 s and 60 s compared with those in their 40 s and 50 s, respectively. Occlusal strength decreases in elderly persons [45], but changes in the occlusal strength of individuals between the ages of 20 and 70 remain unknown. This age-specific trend revealed that the total number of teeth decreased after the age of 40, and that individuals in their 70 s had an average of 20.5 teeth. Therefore, a decrease in the number of teeth is unlikely as the sole cause of decreased occlusal force. Since the sharp decline in occlusal force between the 40 s and 50 s corresponded to significant worsening of disease indicators including NCDs, general health status might somewhat affect periodontal tissues in addition to progress of periodontal disease.

We evaluated associations between periodontal disease index, general health index, and occlusal force. However, multiple linear regression analysis (adjusted for age and smoking status) found a significant negative association emerged between PPD and occlusal force among employees with moderate/severe periodontitis, moderate COPD, and ASCVD. An association has been identified between periodontal disease and moderate COPD and ASCVD, with their common pathology being chronic inflammation. There are similarities in COPD and periodontitis disease mechanisms that of dysfunctional neutrophil behaviors, sustained neutrophilic inflammation, and connective tissue loss caused by oral bacteria [48]. Local expression of tumour necrosis factor (TNF), interleukin-1β (IL-1β), and IL-6 due to inflammation triggered by bacteria involved in periodontal disease might promote and exacerbate ASCVD [49]. Chronic inflammations lead to increased PPD and decreased occlusal force.

Furthermore, HbA1c level and occlusal force were significantly and negatively associated with moderate/severe periodontitis, while %FEV_1_ was significantly and positively associated with occlusal force in the groups with IGT. The biological mechanisms underlying the relationship between diabetes and occlusal force remain obscure. We believe that chronic inflammatory conditions affect the function of the periodontal ligament (PDL), which is involved in generating occlusal force. The PDL plays an important role in distributing occlusal force over the alveolar bone and maintaining the positions of teeth, with mechanical forces altering cell viability, proliferation, and differentiation within the PDL itself [50]. Diabetes increases the abundance of PDL osteoclasts, which might be attributed to the increased expression of receptor activator of nuclear factor kappa B ligand (RANKL) in mouse models of diabetes [51]. Moreover, hyperglycaemia is associated with activation of the NF-κB pathway [52]. Pabisch et al. discovered an abnormal bone structure in a diabetes mouse model [53]. Advanced glycation end products (AGEs) accumulate in the blood serum and in cells and tissues during chronic hyperglycaemia in patients with diabetes [54, 55]. The increased frequency of bone fractures among patients with diabetes might be due to deterioration in bone quality mediated by AGEs [56]. These results indicated that diabetes alters bone metabolism, PDL function, and bone structure, and that these mechanisms could explain the association between diabetes and occlusal force. However, this awaits further investigation.

Poudel et al. and Sanchez et al. have advocated the need to introduce systems to improve oral health, educate patients about increased risk of oral complications, and provide advice about good dental practices. [57, 58]. Health disparities that have recently been viewed as problematic are strongly influenced by income and education, which are also important factors that affect general health [59]. Changing a society’s views on issues such as the importance of oral healthcare or the impact of socio-economic factors and healthcare services as they pertain to quality of life is challenging. However, a model that includes regular oral healthcare should be established and vigorously promoted to achieve societal acceptance. The Industrial Safety and Health Law in Japan requires all companies to have their employees undergo annual health checks, but not dental examinations. This health system has the advantage of being accessible to anyone regardless of income, thus facilitating the accumulation of annual health examination data to monitor baseline health status as well as changes over time. The introduction of periodontal health checks into this system might facilitate the detection of dental health issues that could allow for appropriate action to avoid or prevent symptom exacerbation, as well as associated healthcare costs. The HHCC collects employee health examination data from the point of hiring to the point of retirement. Thus, they implement early detection of systemic diseases and other healthcare measures to prevent worsening of pre-existing conditions. We found that maintaining employee health led to better overall corporate productivity. Moreover, a reduction in medical expenses leads to a more sustainable healthcare system. However, Japanese companies do not apply the same vigilance to oral health as physical health checks. We introduced dental examinations at no additional cost in this study. The rate at which the employees presented at dental clinics at that time was 63.6% (2014), with only a few employees aware of the importance of dental examinations. However, continued annual dental examinations subsequently increased this rate to 81.8% (2015), 89.7% (2016), and 85.1% (2017). People in Japan do not generally attend dental clinics unless subjective symptoms appear, with dentistry remaining outside the scope of regular health maintenance. However, this trend is not unique to Japan, since economic status and lack of knowledge about oral healthcare are established barriers to the inclusion of dentistry in regular health checks in other countries [58]. The association between periodontal disease and general health was communicated to the employees in this study via the internal public relations department, which improved the collective understanding of the importance of regular dental health examinations. When a need for dental treatment was apparent, we provided the affected employee with a referral letter for a thorough dental examination. We also established a medical test feedback system to evaluate the extent to which dental treatment contributes to improved health, as well as how it affects health insurance expenditure. Japan has the highest life expectancy in the world, but healthcare costs are increasing due to the expansion of advanced medical care and the aging population. We believe that expanding systems such as oral healthcare will help slow the burden of increasing medical expenses.

This cross-sectional study had some limitations. In this study, PPD and BOP calculated from PPD and BOP were used as periodontal disease indexes in the analysis, and CAL was not measured. Conventionally, periodontal diseases are classified according to CAL. However, in the large-scale periodontal disease medical examination carried out in this study, it was difficult to accurately measure CAL in other places aside from the clinic within limited time. PISA quantifies the inflammatory burden posed by periodontitis, and can be easily and broadly applied [19]. PISA could be considered an alternative periodontal index that represents an individual's periodontal status, and could be widely applied in various periodontal studies [60]. PISA can be calculated with the help of Excel spreadsheets by entering the values of CAL, location of the gingival margin, and PPD as measured on six sites per tooth. We calculated the PESA and PISA using the spreadsheets by entering the values of PPD and BOP in this study. Nessa et al. suggested that the calculation of PISA using CAL always includes measurement errors related to observer, instrument, and the teeth of patients, and that their interactions might lead to imprecise quality with regards to the amount of tissue [19]. Moreover, they discussed that using PPD instead of CAL, i.e. entering PPD into the formula for CAL, will diminish this underestimation. However, measurement of CAL is required to accurately perform periodontal disease classification, and validation is required on the data obtained. HbA1c, age, and smoking were extracted as important confounding factors from the correlation analysis in this study. In the multiple logistic analysis, these were used as covariates to calculate the Odds ratio and SPRC (β). However, other potential confounding factors may include gender, lifestyle habits such as alcohol intake, and drug administration, and the results may differ. The correlation between dental and health indicators does not necessarily imply a causal relationship. In addition to the factors that we analysed herein, other factors influence the association between periodontal disease and general health. Therefore, long-term follow-up studies are needed to establish causal relationships. This study included a limited group of corporate employees that included only 10.6% women; therefore, our findings were biased towards men. Thus, long-term follow-up studies involving a larger female cohort are needed to fully determine causal relationships.

## Conclusions

This cross-sectional study introduced dental examinations into the regular health check program for employees of Hitachi Ltd., and identified an association between periodontal disease and elevated risk of NCDs, especially obesity, diabetes, IGT, COPD, ASCVD. In addition, our results revealed that changes in occlusal force varied with age, and that future health issues could be accurately predicted from the results of routine dental examinations. Including dental examinations in compulsory corporate health checks facilitates the early detection of periodontal disease as well as therapeutic interventions. Closer medical-dental collaborations are important, and these should help to build a comprehensive regional healthcare system that maintains and promotes general health.

## Supplementary Information

**Additional file 1: Table S7**. Relationship between periodontal or general health indices and occlusal force by multiple regression analysis. Reference Model 1 (M1) is based on healthy employees with HbA1c level < 5.7. Model 2 (M2) is M1 adjusted for age. Model 3 (M3) is M1 adjusted for age and smoking status. SPRC (β), standardized partial regression coefficient. CI, confidence interval. Values with p < 0.05 were considered statistically significant. Values in bold are statistically significant.

## Data Availability

The dataset used and analysed during this study is available upon reasonable request from the corresponding author.

## Abbreviations

ASCVD: Atherosclerosis cardiovascular disease
BMI: Body mass index
BOP: Bleeding on probing
CAVI: Cardio-ankle vascular index
COPD: Chronic obstructive pulmonary disease
FBG: Fasting blood glucose
FEV_1_: Forced expiratory volume in 1 s
FIB4: Liver fibrosis index
HbA1c: Haemoglobin A1c
FVC: Forced vital capacity
HDL: High-density lipoprotein
LDL: Low-density lipoprotein
PESA: Periodontal epithelial surface area
PISA: Periodontal inflamed surface area
PPD: Probing pocket depth
