# Supplementary material for: A cross-sectional study of relationships between periodontal disease and general health: The Hitachi Oral Healthcare Survey
Source: BMC Oral Health. 2021 Dec 15;21:644. doi: 10.1186/s12903-021-01990-6 (PMC8672581; doi:10.1186/s12903-021-01990-6)
Supplement: Supplementary file 1 — Additional file 1: Table S7. Relationship between periodontal or general health indices and occlusal force by multiple regression analysis. Reference Model 1 (M1) is based on healthy employees with HbA1c level < 5.7. Model 2 (M2) is M1 adjusted for age. Model 3 (M3) is M1 adjusted for age and smoking status. SPRC (β), standardized partial regression coefficient. CI, confidence interval. Values with p < 0.05 were considered statistically significant. Values in bold are statistically significant. [file 12903_2021_1990_MOESM1_ESM.docx]

**Table 7** Relationship between periodontal or general health indices and occlusal force by multiple regression analysis

|  |  | Occlusal force (N) | | | | | | | | | | | | | | | | | | | | | | | |
| --- | --- | --- | --- | --- | --- | --- | --- | --- | --- | --- | --- | --- | --- | --- | --- | --- | --- | --- | --- | --- | --- | --- | --- | --- | --- |
|  |  | Moderate/Severe  periodontitis  (*n* = 287) | | | | Obesity  (*n* = 266) | | | | Diabetes  (*n* = 71) | | | | IGT  (*n* = 96) | | | | Moderate COPD  (*n* = 367) | | | | Arteriosclerosis  (*n* = 79) | | | |
| Variables | Model | 95% CI | | β | *P* | 95% CI | | β | *P* | 95% CI | | β | *P* | 95% CI | | β | *P* | 95% CI | | β | *P* | 95% CI | | β | *P* |
| PPD  (mm) | M1 | -122.9 | -39.78 | - 0.224 | **< 0.001** | -80.32 | 5.19 | - 0.107 | 0.085 | -266.4 | -24.72 | - 0.282 | **0.019** | -167.4 | -0.61 | - 0.204 | **0.048** | -96.80 | -19.41 | - 0.153 | **0.003** | -183.8 | -62.79 | - 0.424 | **< 0.001** |
|  | M2 | -106.8 | -25.28 | - 0.182 | **0.002** | -61.65 | 18.22 | - 0.062 | 0.285 | -224.0 | 6.58 | - 0.210 | 0.064 | -157.8 | 0.31 | - 0.191 | 0.051 | -89.16 | -12.76 | - 0.134 | **0.009** | -181.1 | -57.11 | - 0.410 | **< 0.001** |
|  | M3 | -110.9 | -27.94 | - 0.191 | **0.001** | -59.61 | 20.83 | - 0.055 | 0.343 | -214.3 | 20.40 | - 0.188 | 0.104 | -159.3 | 1.19 | - 0.192 | 0.053 | -91.83 | -14.20 | - 0.139 | **0.008** | -183.1 | -54.65 | - 0.409 | **< 0.001** |
| BMI  (kg/m^2^) | M1 | -9.23 | 5.19 | - 0.033 | 0.577 | -8.69 | 9.80 | 0.007 | 0.906 | -4.27 | 27.00 | 0.174 | 0.152 | -8.91 | 21.10 | 0.084 | 0.422 | -14.37 | 2.56 | - 0.072 | 0.171 | -13.59 | 19.66 | 0.042 | 0.717 |
|  | M2 | -11.40 | 2.59 | - 0.071 | 0.216 | -18.10 | -0.27 | - 0.122 | **0.044** | -23.06 | 12.36 | - 0.082 | 0.548 | -20.82 | 10.58 | - 0.070 | 0.519 | -14.22 | 2.37 | - 0.072 | 0.161 | -14.40 | 18.80 | 0.030 | 0.792 |
|  | M3 | -11.48 | 2.55 | - 0.072 | 0.211 | -18.09 | -0.30 | - 0.122 | **0.043** | -27.25 | 9.02 | - 0.140 | 0.319 | -21.56 | 10.46 | - 0.076 | 0.493 | -14.38 | 2.31 | - 0.073 | 0.156 | -14.95 | 18.40 | 0.024 | 0.837 |
| HbA1c  (%) | M1 | -90.16 | -17.49 | - 0.171 | **0.004** | -49.81 | 12.17 | - 0.074 | 0.233 | -46.64 | 76.54 | 0.059 | 0.630 | -56.23 | 38.98 | - 0.037 | 0.720 | -71.72 | -7.54 | - 0.126 | **0.016** | -105.0 | 36.76 | - 0.110 | 0.341 |
|  | M2 | -76.13 | -5.03 | - 0.129 | **0.025** | -40.91 | 16.58 | - 0.048 | 0.406 | -60.81 | 54.67 | - 0.012 | 0.916 | -62.40 | 29.01 | - 0.075 | 0.453 | -58.76 | 6.11 | - 0.084 | 0.111 | -113.1 | 29.07 | - 0.135 | 0.243 |
|  | M3 | -77.71 | -5.97 | - 0.133 | **0.022** | -39.70 | 18.01 | - 0.043 | 0.460 | -60.92 | 53.83 | - 0.014 | 0.902 | -62.61 | 28.40 | - 0.074 | 0.457 | -59.95 | 5.63 | - 0.086 | 0.104 | -110.3 | 35.27 | - 0.121 | 0.308 |
| FBG (mg/dL) | M1 | -3.25 | -0.56 | - 0.164 | **0.006** | -1.69 | 0.63 | - 0.056 | 0.368 | -1.53 | 3.13 | 0.084 | 0.494 | -2.26 | 2.54 | 0.012 | 0.907 | -2.33 | 0.01 | - 0.102 | 0.051 | -4.31 | 0.42 | - 0.186 | 0.106 |
|  | M2 | -2.64 | 0.01 | - 0.113 | 0.052 | -1.14 | 1.02 | - 0.006 | 0.915 | -1.84 | 2.50 | 0.034 | 0.765 | -2.31 | 2.25 | - 0.003 | 0.980 | -1.82 | 0.54 | - 0.056 | 0.288 | -4.27 | 0.44 | - 0.183 | 0.109 |
|  | M3 | -2.72 | -0.03 | - 0.118 | **0.045** | -1.07 | 1.12 | 0.002 | 0.967 | -1.54 | 2.86 | 0.069 | 0.548 | -2.31 | 2.33 | 0.001 | 0.993 | -1.85 | 0.53 | - 0.058 | 0.279 | -4.20 | 0.61 | - 0.171 | 0.141 |
| % FEV_1_ | M1 | 0.28 | 8.16 | 0.125 | **0.036** | 2.49 | 12.94 | 0.180 | **0.004** | 7.77 | 28.60 | 0.392 | **< 0.001** | 5.53 | 22.44 | 0.326 | **0.001** | -3.45 | 6.44 | 0.031 | 0.553 | -3.12 | 8.99 | 0.112 | 0.337 |
|  | M2 | -2.29 | 5.65 | 0.050 | 0.405 | -2.43 | 8.02 | 0.065 | 0.293 | 1.10 | 23.51 | 0.265 | **0.032** | 0.29 | 18.71 | 0.222 | **0.043** | -4.75 | 5.04 | 0.003 | 0.954 | -3.27 | 8.83 | 0.106 | 0.363 |
|  | M3 | -2.28 | 5.68 | 0.050 | 0.401 | -2.69 | 7.82 | 0.060 | 0338 | -1.20 | 22.93 | 0.234 | 0.077 | 0.29 | 19.21 | 0.227 | **0.044** | -4.75 | 5.06 | 0.003 | 0.951 | -3.81 | 8.60 | 0.091 | 0.443 |
| CAVI | M1 | -87.19 | -17.99 | - 0.216 | **0.003** | -86.83 | -12.20 | - 0.193 | **0.010** | -116.6 | 10.92 | - 0.241 | 0.102 | -119.3 | 6.73 | - 0.229 | 0.079 | -61.19 | -4.02 | - 0.143 | **0.026** | -179.1 | -6.29 | - 0.240 | **0.036** |
|  | M2 | -48.84 | 45.09 | - 0.008 | 0.937 | -20.04 | 73.55 | 0.104 | 0.261 | -69.06 | 83.24 | 0.032 | 0.852 | -75.30 | 86.47 | 0.023 | 0.890 | -35.10 | 43.64 | 0.019 | 0.831 | -172.8 | 7.80 | - 0.213 | 0.073 |
|  | M3 | -48.08 | 45.22 | - 0.060 | 0.952 | -20.92 | 72.76 | 0.101 | 0.276 | -67.73 | 86.09 | 0.042 | 0.811 | -75.30 | 86.47 | 0.023 | 0.889 | -34.66 | 44.43 | 0.021 | 0.808 | -176.6 | 4.60 | - 0.222 | 0.062 |

Reference Model 1 (M1) is based on healthy employees with HbA1c level < 5.7. Model 2 (M2) is M1 adjusted for age. Model 3 (M3) is M1 adjusted for age and smoking status. SPRC (β), standardized partial regression coefficient. CI, confidence interval. Values with *p* < 0.05 were considered statistically significant. Values in bold are statistically significant**.**
